# Celocentesis: Current Evidence and Future Directions

**DOI:** 10.3390/jcm15062196

**Published:** 2026-03-13

**Authors:** Fabiana Cecchini, Silvia Visentin, Antonino Giambona, Margherita Vinciguerra, Francesco Picciotto, Alessandra Andrisani, Matteo Cassina, Ambrogio Pietro Londero, Kypros Nicolaides, Erich Cosmi

**Affiliations:** 1 Maternal Fetal Medicine Unit, Department of Women’s and Children’s, School of Medicine, University of Padova, 35131 Padova, Italy; silvia.visentin.1@unipd.it (S.V.); erich.cosmi@unipd.it (E.C.); 2 UOSD Diagnostica Molecolare Malattie Rare Ematologiche, AOOR Villa Sofia Cervello, 90100 Palermo, Italy; a.giambona@villasofia.it (A.G.); m.vinciguerra@villasofia.it (M.V.); 3 Unit of Fetal Medicine and Prenatal Diagnosis, Azienda Ospedaliera Ospedali Riuniti Villa Sofia Cervello, 90146 Palermo, Italy; francescopicciotto64@gmail.com; 4 Department of Women’s and Children’s Health, University of Padova, 35128 Padova, Italy; alessandra.andrisani@unipd.it; 5 Clinical Genetics Unit, Department of Women’s and Children’s Health, University of Padova, 35131 Padova, Italy; matteo.cassina@unipd.it; 6 Obstetrics and Gynecology Unit, IRCCS Istituto Giannina Gaslini, 16147 Genova, Italy; ambrogio.londero@gmail.com; 7 Department of Neuroscience, Rehabilitation, Ophthalmology, Genetics, Maternal and Infant Health, University of Genoa, 16132 Genova, Italy; 8 Harris Birthright Research Centre for Fetal Medicine, King’s College Hospital, London SE5 9RS, UK; kypros@fetalmedicine.com

**Keywords:** celocentesis, coelocentesis, coelomic fluid sampling, coelomic cavity sampling, first-trimester coelomic fluid, prenatal diagnosis, coelomic fluid, early pregnancy

## Abstract

**Background/Objectives:** This study aimed to review current evidence on celocentesis as an early invasive prenatal diagnostic technique, focusing on its clinical applications, diagnostic accuracy, safety profile, and future perspectives in modern fetal medicine. **Methods:** A narrative review of the literature was conducted through PubMed and Scopus databases up to October 2025. Studies reporting original data on celocentesis—including prospective studies, case reports, and case series—were included. Relevant outcomes were feasibility, safety, and diagnostic accuracy. **Results:** Since its first description in 1993, celocentesis has been successfully performed between 6 and 9 weeks’ gestation in several small case series. Improvements in ultrasound resolution and molecular analysis techniques have significantly enhanced its reliability. In specialized centers using dedicated fetal cell selection and contamination-control workflows, analytical diagnostic success for selected monogenic conditions exceeds 99%. Reported miscarriage rates are comparable to what is expected at a very early gestational age (10% of all clinically recognized pregnancies). The procedure remains mainly experimental, with no standardized protocols or large multicentric validation. **Conclusions:** Celocentesis is the earliest available technique for prenatal genetic diagnosis. While promising, its clinical implementation requires further standardization, comprehensive operator training, and robust evidence from prospective studies regarding its safety and diagnostic reliability.

## 1. Introduction

In recent decades, prenatal diagnosis has undergone a significant transformation, paralleling advances in ultrasound and molecular biology techniques. Alongside traditional invasive methods such as chorionic villous sampling (CVS) and amniocentesis, there has been growing interest in approaches that can provide genetic and molecular information at an earlier stage of embryogenesis. In this context, celocentesis is a promising sampling technique. Celocentesis, first described in 1993, involves ultrasound-guided aspiration of fluid from the coelomic cavity [1]. The coelomic cavity is an extraembryonic space that forms around the 5th week of gestation and surrounds the embryo until the amniotic sac expands at around the 10th week [1]. It contains embryonic cells and an electrolyte-rich fluid fraction that is also rich in vitamins, amino acids, proteins, and metabolites [1,2,3]. The ability to access this cavity safely and selectively has opened new perspectives for both early genetic diagnosis and the physiological study of human development. Pioneering studies conducted in the 1990s demonstrated the technical feasibility of the procedure and the presence of viable fetal-derived cells in the coelomic fluid. However, limitations in culture methods and difficulty distinguishing between maternal and embryonic contributions initially represented an obstacle to its clinical application [1,4,5,6]. With the application of fluorescence in situ hybridization (FISH) and polymerase chain reaction (PCR) techniques, celocentesis has shown diagnostic potential comparable to, or even greater than, that of invasive procedures performed later in pregnancy [7,8,9,10]. Monogenic diseases have been diagnosed by celocentesis, and the potential of genome-wide chromosomal analysis has also been explored [11,12,13]. This narrative review was conducted to critically summarize current knowledge and discuss the potential future clinical and research applications of celocentesis, focusing on its clinical applications, diagnostic accuracy, safety profile, and future perspectives in modern fetal medicine.

## 2. Materials and Methods

A non-systematic review was conducted in accordance with the Scale for the Assessment of Narrative Review Articles (SANRA) for the quality assessment of narrative review articles [14]. A study protocol was developed before the review began. Literature searches were conducted in October 2025 in MEDLINE/PubMed, Scopus, and the Cochrane Library using the search terms celocentesis, coelocentesis, coelomic fluid sampling, coelomic cavity sampling, and first-trimester coelomic fluid. Records were limited to the English language. In addition, reference lists from included original papers and review articles were searched to identify any other relevant studies. As this was a descriptive review, no quantitative synthesis was performed; the findings were analyzed narratively, focusing on the evolution of the procedure, its clinical applications, and its safety profile [15]. The study population included singleton pregnancies that underwent celocentesis for genetic testing. Original research articles on celocentesis, case reports, case series, and reviews were included. Letters to the editor/comments, duplicates, studies in which it was impossible to retrieve information on the selected outcomes, abstracts or conference proceedings without full text, non-peer-reviewed materials, and articles in a language other than English were excluded from the analysis. Animal studies were also excluded. Each study was assessed by one reviewer (F.C.) using predetermined inclusion/exclusion criteria. A second reviewer (S.V.) was available to resolve uncertain cases. An initial screening of titles and abstracts was performed, followed by a detailed full-text review. The predefined outcomes were feasibility, safety, and the diagnostic accuracy of the procedure.

Because several publications originated from the same centers and some reports represented updates or extensions of previously published series, we specifically assessed potential cohort overlap by comparing recruitment periods, clinical settings, sample size, and inclusion criteria. When overlap was identified or strongly suspected, we did not treat these papers as independent datasets and avoided summing participants across overlapping reports. For each overlapping series, data were extracted preferentially from the most comprehensive and/or most recent publication for key outcomes (feasibility, diagnostic performance, and complications), while earlier reports were used only for complementary methodological information or outcomes not reported elsewhere.

## 3. Results

The initial search retrieved 333 records; 270 were excluded (duplicates, animal studies, non-English language, or insufficient information on the prespecified outcomes), leaving 63 studies for full-text review. After full-text assessment, 33 articles were excluded because their reported outcomes were not relevant, resulting in 30 studies included in the narrative review (Table 1).

The included studies were published between 1993 and 2024 and were mainly contributed by two research groups: the Greek group led by Makrydimas and the Italian group coordinated by Giambona. A total of 24 prospective studies and 6 case reports were included. Celocentesis was performed between 6 and 12 weeks’ gestation, with the largest proportion of procedures (45.7%) carried out between 7 and 9 + 6 weeks; only one study used embryonic crown–rump length (<18 mm) rather than gestational age as the reference parameter. Most early cohorts involved patients already scheduled for termination of pregnancy or dilation and curettage for missed miscarriage, whereas more recent studies increasingly describe elective procedures in ongoing pregnancies in an outpatient setting (9/24 prospective studies, 37.5%).

### 3.1. Technical Feasibility and Sampling

Across published series (in total 1750 procedures), the procedure was generally technically feasible, with failure to access the coelomic cavity reported only rarely (Table 2). In a large prospective safety series, coelomic fluid was successfully obtained in all but one case (access prevented by a large anterior wall fibroid), and in 98% of cases, sampling was achieved with a single needle insertion [16]. In a study assessing feto-maternal bleeding, coelomic fluid was aspirated successfully in all 17 cases (median volume 1.6 mL, range 0.5–3.0), with no significant change in maternal serum AFP after the procedure [17].

### 3.2. Sample Cellularity and Maternal Cell Contamination (MCC)

Several studies highlight that coelomic fluid is pauci-cellular and highly variable. In one study quantifying cellularity at 8–9 weeks, cell density ranged from 0 to 10,600 cells/mL; FISH failed in three cases due to absent/very low cell numbers, and in two additional cases, FISH demonstrated a high count of maternal cells despite the fluid not appearing blood-stained, supporting the need for systematic exclusion of significant MCC when interpreting results [18]. Similarly, an early PCR feasibility study achieved successful multiplex real-time PCR in 71% (10/14) of samples, but “reliable” prenatal diagnoses (successful PCR and no marked MCC) in only 58% (8/14) [19].

### 3.3. Diagnostic Approaches and Concordance

Across the included literature, PCR-based and other molecular methods were the most frequently reported diagnostic approaches, followed by standard cytogenetics and FISH-based testing. Overall, 25.4% of coelomic samples underwent PCR or other molecular genetic analyses, while 17.1% were analyzed using conventional cytogenetics and/or FISH. Molecular analysis was reported in nine studies, with an overall diagnostic accuracy of 99% (Table 3) and near-complete concordance with the reference diagnosis. In the largest hemoglobinopathy series using embryo–fetal erythroid cell selection strategies, 302 cases were reported with fetal genotypes fully concordant with the reference diagnosis across different contamination strata, including cases requiring immunomagnetic selection or micromanipulation [20].

Similarly, in a large prospective investigation (385 pregnancies) focusing on coelomic fluid characteristics and molecular feasibility, the authors highlighted the importance of dedicated laboratory workflows for fetal cell selection in paucicellular samples [10].

### 3.4. Safety Outcomes

Controlled evidence on short-term safety is limited and heterogeneous. In a small controlled series (20 celocentesis vs. 100 matched controls, all performed before planned termination), miscarriage during follow-up occurred in 25% of cases after celocentesis versus 5% in controls [21]. In contrast, in a subsequent controlled study (108 celocentesis vs. 339 controls), miscarriage rates were 4.7% and 2.7%, respectively, with the authors estimating a procedure-related fetal loss of about 2% [16].

However, differences in study design, gestational age, and clinical context limit direct comparability between series.

## 4. Discussion

### 4.1. Biological Rationale

The coelomic cavity develops around the fourth week of gestation and persists until approximately the twelfth week. It surrounds the embryo and the amniotic cavity, reaching its maximum volume between the seventh and ninth weeks of gestation. Coelomic fluid is an ultrafiltrate of maternal serum, rich in proteins, amino acids, glucose, urea, and many other molecules that are transferred, through a pressure gradient system, to the amniotic sac and the embryo [2,3]. Coelomic fluid also contains fetal cells. Within the coelomic cavity, the yolk sac floats freely. Hematopoietic progenitors are initially produced by the mesodermal layer of the yolk sac and then complete their development in the embryonic bloodstream [35].

### 4.2. Execution Technique

The external genitalia and vagina should be carefully cleaned with an antiseptic solution. Then, transvaginal sonography with a sterile, 5-MHz ultrasound probe should be performed. It is important to measure the fetal crown–rump length and fetal heart rate and to identify the amniotic membrane, coelomic space, and yolk sac. A 20 G needle, attached to a guide on the transducer, should be inserted transvaginally into the coelomic cavity to aspirate fluid. No local or general anesthesia is needed. The initial 0.2 mL sample can be discarded to prevent contamination from maternal tissue, and then an additional 1 mL of fluid should be aspirated for testing [7].

### 4.3. Technical Challenges

Specific technical skills, a clear ultrasound view, and strict laboratory protocols are required to perform celocentesis. Identifying and correctly accessing the coelomic cavity is the first key step. The procedure can only be performed within a very short time frame, between the 7th and 9th weeks of gestation, a period during which the size of the cavity and the position of the embryonic sac vary significantly according to uterine orientation and the site of implantation of the pregnancy. The presence of a thickened myometrium, uterine retroversion, or an eccentrically located gestational sac can make needle sampling more difficult. At this stage, the quality of the ultrasound image and the operator’s experience are crucial for minimizing the risk of sampling failure [7]. The concentration of fetal cells within the sample is typically low and variable. The integration of inverted microscopy and micromanipulation has significantly improved the differentiation between maternal and fetal cells, thereby enabling their selective aspiration. This laboratory step remains the primary factor determining diagnostic accuracy [10]. From a laboratory perspective, the amount of DNA obtained is an additional challenge because the amount of genetic material is extremely small and often fragmented. Therefore, a highly sensitive and specific workflow is necessary to minimize amplification errors and ensure reproducible results [16].

### 4.4. Safety and Complications

Over more than 30 years of research, celocentesis has shown a specific safety profile when performed in expert centers. Early studies in the 1990s primarily focused on the technical feasibility and immediate outcomes of the procedure. The results of these early studies were inconsistent. In 1997, Makrydimas et al. systematically evaluated maternal and fetal safety, finding no major complications such as miscarriage, hemorrhage, or infection when the procedure was performed with fine needles (20–22 G) under sterile conditions [17]. However, in the same year, Ross et al. reported an increased risk of miscarriage (25% in the population undergoing celocentesis versus 5% in the control group) [21]. Subsequent prospective studies have not confirmed this finding. They reported a low risk of maternal–fetal complications, emphasizing that if access to the coelomic cavity occurs before the fusion of the chorioamniotic membranes, the risk of rupture or loss of amniotic fluid is lower than with later invasive techniques [7,16]. The rate of spontaneous abortion after celocentesis is similar to the baseline risk at the same gestational age [11]. However, because early losses are common and frequently unrelated to procedures, disentangling procedure-related effects from background loss at very early gestational ages remains difficult.

Sample quality and operator experience are key factors for safety and diagnostic success, with aspiration failures more often caused by technical limitations than clinical complications [19,22].

Overall, the interpretation of miscarriage rates after celocentesis is complicated by the substantial background risk of pregnancy loss at 6–10 weeks’ gestation and by differences in study design (elective ongoing pregnancies vs. procedures performed before planned termination or after missed miscarriage). Available controlled data are limited and heterogeneous, and current series—although reassuring in expert hands—cannot exclude a small procedure-related risk. Consequently, statements on safety should be considered provisional until large, prospective, multicenter studies using standardized definitions, appropriate control groups, and systematic follow-up are available [8,9,10,11,20,23].

An additional practical implication of performing invasive sampling at very early gestational ages is that a proportion of pregnancies will subsequently undergo spontaneous loss unrelated to the procedure. Therefore, some procedures may be carried out in pregnancies that would later be declared nonviable, independent of the intervention. This underscores the importance of confirming viability immediately prior to sampling, discussing background early gestational loss risk during counseling, and considering short-interval reassessment when viability is uncertain.

### 4.5. Historical Evolution

**Figure 1:**
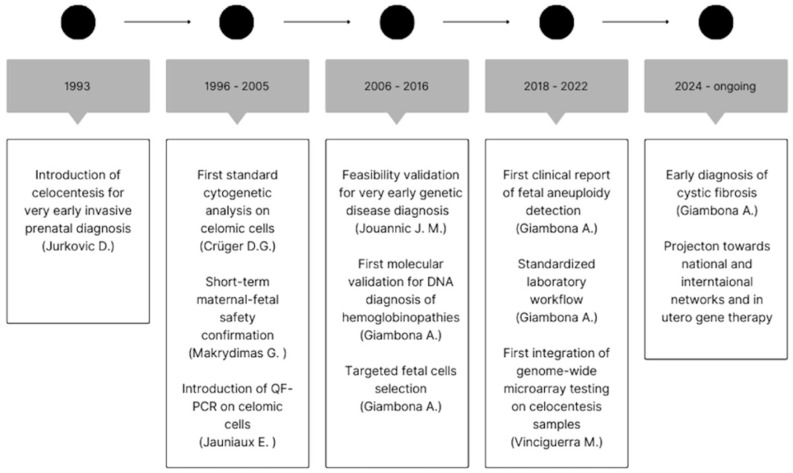
Summary table of the historical development and main milestones of celocentesis.

Figure 1 summarizes the main milestones in the development of celocentesis from its first introduction in 1993 to its current clinical and translational applications. The timeline highlights the progressive evolution of the technique, including the first cytogenetic and molecular analyses on coelomic cells, validation of its feasibility and safety for very early prenatal diagnosis, refinement of fetal cell selection strategies, subsequent expansion toward early diagnosis of monogenic disorders and the establishment of standardized workflows, supporting the transition of celocentesis from an experimental procedure toward broader clinical implementation and future integration into national and international collaborative networks.

### 4.6. Diagnostic Application

Since its introduction, celocentesis has evolved from an experimental, invasive prenatal diagnostic tool into a reliable and useful clinical tool. The first feasibility studies showed that ultrasound-guided puncture of the coelomic cavity could be performed safely between the sixth and ninth weeks of gestation. Thin needles (20–22 G) were used. The procedure was successful in over 90% of cases, with the coelomic cavity easily identified before fusion of the chorioamniotic membranes [1,24]. In subsequent years, the presence of intact embryo–fetal cells in the coelomic fluid, suitable for cytogenetic and molecular analysis, was confirmed. The first diagnostic applications included FISH analysis and fetal sex determination by PCR, demonstrating high concordance with results from chorionic villus sampling or amniocentesis. However, limited cell yield and maternal contamination remained critical factors, resulting in variable diagnostic success rates [5,17]. Improvements in sample-handling techniques expanded the diagnostic applications of the test. Quantitative fluorescent PCR (QF-PCR) for the rapid detection of aneuploidies was first reported by Jauniaux in 2003 [22]. The refinement of FISH and the introduction of single-gene analysis confirmed the validity of coelomic fluid as an early source of fetal DNA [25,26]. A further improvement came with the optimization of embryo–fetal erythroid cell selection and the development of protocols for DNA extraction and amplification, achieving diagnostic success rates of over 99% for hemoglobinopathies and other monogenic diseases [8,9,20,23]. In recent years, celocentesis has entered the era of molecular genomics. The successful application of next-generation sequencing (NGS) and array-CGH techniques to coelomic DNA has enabled the diagnosis of monogenic disorders such as β-thalassemia, cystic fibrosis, Hb Lepore, and Cockayne syndrome. The diagnostic accuracy reported in these studies is comparable to that of traditional invasive methods, with the advantage that samples can be obtained one to four weeks earlier [10,11,13]. Reported diagnostic success rates of > 99% should be interpreted as analytical performance. Diagnostic reliability is strictly dependent on dedicated workflows for fetal selection, contamination control, and operator expertise.

### 4.7. Molecular and Genomic Advances

Over the past 20 years, advances in molecular biology techniques have greatly improved the diagnostic capabilities of celocentesis, transforming it from an experimental method into a viable option for more complex genetic testing. Since the first attempts at in situ hybridization and qualitative PCR in the 1990s, there has been a gradual shift towards highly sensitive and specific methods capable of analyzing minimal amounts of fetal DNA and reliably distinguishing embryo–fetal material from maternal material. A turning point was the introduction of quantitative fluorescent PCR (QF-PCR) and fluorescence in situ hybridization (FISH) on isolated coelomic cells, which made it possible to validate the presence of authentic fetal DNA and obtain the first diagnoses of early aneuploidies [22,26,27]. Subsequently, improvements in extraction, filtration, and cell-selection techniques, especially the enrichment of fetal erythroid cells, enhanced sample purity and increased the diagnostic yield of molecular analyses [9]. These methods now enable the detection of microdeletions, duplications, and point variants with accuracy similar to that of chorionic villus sampling and amniocentesis. Recent studies have shown that reliable results can be obtained from very small amounts of DNA, including the detection of monogenic disorders (such as β-thalassemia, cystic fibrosis, Hb Lepore, and Cockayne syndrome) and chromosomal abnormalities (such as trisomies and complex structural variants) at gestational ages of less than 10 weeks [11,12,13,28]. A further advance has been the development of dedicated laboratory workflows capable of managing the entire process from sample collection to the generation of genetic reports. These protocols, introduced and standardized in Italy by Giambona’s group, integrate quality control, automated extraction, and bioinformatic validation of sequencing data [13].

### 4.8. Comparison with Other Prenatal Diagnostic Methods

Celocentesis is performed earlier than non-invasive prenatal testing (NIPT) and traditional invasive testing. It is the only invasive procedure that can be performed in the early first trimester, providing direct access to embryo–fetal genetic material weeks before chorionic villus sampling, which is usually performed between 11 and 13 weeks [36]. CVS analyzes chorionic tissue from the trophoblast. Amniocentesis identifies and analyzes fetal cells in amniotic fluid; it can be performed after the 15th week of gestation [36]. Cell-free DNA testing in maternal blood offers high sensitivity for certain common aneuploidies, but it is a screening method and requires invasive confirmation [37].

This distinction may be clinically relevant because celocentesis accesses embryo–fetal cells rather than placental tissue. Unlike placental-based approaches such as CVS and cfDNA screening, it may theoretically reduce concerns related to confined placental mosaicism, although this potential advantage requires further confirmation in larger studies.

From a diagnostic perspective, celocentesis allows very early genetic analyses to be performed, as early as the 7th week [7]. To date, this technique has enabled the diagnosis of a wide range of conditions, especially monogenic diseases (β-thalassemia, cystic fibrosis, Hb Lepore, Cockayne syndrome) [13,28]. Compared to CVS and amniocentesis, celocentesis offers a significant time advantage, allowing parents to obtain a definitive genetic diagnosis at a stage when decision-making and treatment options are broader [10]. The diagnostic performance reported in the latest case studies shows a success rate of over 99%, with analytical accuracy comparable to standard techniques [10].

From a risk perspective, CVS and amniocentesis are well-established procedures with low complication rates in contemporary practice, whereas celocentesis has a much smaller evidence base, and safety estimates are more vulnerable to center effects and early gestational background loss.

In terms of availability, CVS/amnio and NIPT are widely accessible in most healthcare systems, whereas celocentesis currently remains limited to a few highly specialized centers because of the narrow gestational window and the requirement for dedicated laboratory workflows to manage paucicellular samples and maternal cell contamination.

The learning curve for celocentesis does not appear to be prohibitive: while it requires appropriate training and coordination between the operator and the laboratory, centers already experienced in transvaginal invasive procedures can typically incorporate the technique without a particularly complex learning pathway. Cost considerations are also relevant: overall procedural costs are broadly comparable to those of CVS and amniocentesis, and the main cost drivers are largely related to downstream genetic/molecular analyses, as in standard invasive diagnostic pathways. Additional laboratory steps (e.g., fetal cell selection) may be required in selected cases, but these do not necessarily translate into higher overall costs compared with established invasive procedures.

### 4.9. Strengths and Limitations of the Available Evidence

The evidence base for celocentesis remains limited by small sample sizes, substantial heterogeneity in study design and reporting, the lack of pooled quantitative analyses, and scarce information on long-term outcomes. Moreover, a large proportion of the published experience originates from only two research groups that have led most of the recent investigations (Palermo group: 9/30 studies; Ioannina group: 7/30 studies). This concentration of expertise raises the possibility of center-specific performance effects and reduces the external validity of reported feasibility, diagnostic performance, and complication rates.

Despite these limitations, feasibility findings are broadly consistent across the available literature, suggesting that—when performed by experienced operators within dedicated laboratory workflows—celocentesis can be technically achievable and may provide a valuable route to embryonic material at very early gestational ages. However, the generalizability of current performance estimates to less experienced settings remains uncertain and should be interpreted cautiously until large, prospective multicenter studies using standardized protocols and uniform outcome definitions become available.

### 4.10. Future Directions

Celocentesis represents a possible application window for fetal gene therapy, which is a promising approach to treat severe genetic disorders by intervening during a critical developmental period. The fetal window provides several advantages, including improved tissue accessibility, immune tolerance, and prevention of permanent organ injury before birth [38].

## 5. Conclusions

Celocentesis is a technically feasible, reproducible, and diagnostically reliable procedure. Recent advances in molecular biology and genetics have expanded the potential uses of celocentesis in prenatal diagnosis. The high analytical sensitivity of next-generation sequencing (NGS) techniques and improved fetal cell selection have addressed many of the method’s historical limitations, making celocentesis a promising approach for early genetic diagnosis. It provides genetic information earlier than traditional techniques such as CVS or amniocentesis. Although it does not replace conventional invasive methods, celocentesis offers a new clinical option for couples at high genetic risk who are seeking a definitive diagnosis early in pregnancy. At present, celocentesis should be considered a highly specialized procedure limited to selected cases in expert centers. Multicenter interlaboratory standardization and integration with molecular genetic platforms are crucial for its future routine clinical implementation.

## Figures and Tables

**Table 1:** Characteristics of included studies.

Study	Country	Study Design	Gestational Age	Technique Details
Jurkovic D (1993) [1]	UK	Prospective	6 w–12 w	20 G needle
Crüger DG (1996) [4]	Denmark	Prospective	6 w–10 w	18 G needle
Findlay I (1996) [5]	UK	Prospective	7 w–10 w	Not Reported
Makrydimas G (1997) [6]	Greece	Prospective	6 w–11 w	20 G needle
Makrydimas G (2004) [7]	Greece	Prospective	7 w–8 w	20 G needle
Giambona A (2011) [8]	Italy	Prospective	7 w–9 w	20 G needle
Giambona A (2016) [9]	Italy	Prospective	7 w–10 w	20 G needle
Giambona A (2022) [10]	Italy	Prospective	7 w–10 w	20 G needle
Makrydimas G (2020) [11]	Italy	Prospective	<18 mm	20 G needle
Vinciguerra M (2022) [12]	Italy	Case report	8 w–9 w	20 G needle
Giambona A (2024) [13]	Italy	Prospective	9 w–10 w	20 G needle
Makrydimas G (2002) [16]	Greece	Prospective	6 w–10 w	20 G needle
Makrydimas G (1997) [17]	Greece	Prospective	7 w–9 w	20 G needle
Jouannic JM (2006) [18]	France	Prospective	8 w–9 w	20 G needle
Jouannic JM (2008) [19]	France	Prospective	8 w–9 w	20 G needle
Giambona A (2016) [20]	Italy	Prospective	7 w–10 w	20 G needle
Ross JA (1997) [21]	UK	Prospective	6 w–10 w	20 G needle
Jauniaux E (2003) [22]	UK	Prospective	6 w–9 w	20 G needle
Giambona A (2018) [23]	Italy	Prospective	7 w–10 w	20 G needle
Crüger DG (1997) [24]	Denmark	Case report	9 w–10 w	Not Reported
Makrydimas G (2004) [25]	Greece	Prospective	7 w–9 w	20 G needle
Chatzimeletiou K (2005) [26]	UK	Prospective	6 w–9 w	20 G needle
Chatzimeletiou K (2006) [27]	UK	Case report	9 w–10 w	20 G needle
Giambona A (2022) [28]	Italy	Prospective	9 w	20 G needle
Makrydimas G (2006) [29]	Greece	Prospective	7 w–10 w	20 G needle
Tonni G (2009) [30]	Italy	Case report	10 w	20 G needle
Tonni G (2009) [31]	Italy	Case report	10 w	20 G needle
Tonni G (2016) [32]	Italy	Case report	11 + 3 w	20 G needle
Aiello (2018) [33]	Italy	Prospective	7 w–10 w	20 G needle
Giambona A (2022) [34]	Italy	Prospective	9 w	20 G needle

**Table 2:** Sampling feasibility and factors associated with a reduced likelihood of successful sampling.

Study	Sampling Success (n/N, %)	Sampling Failure (n/N, %)	Constraints
Jurkovic D (1993) [1]	100/100 (100%)	0	0
Crüger DG (1996) [4]	30/30 (100%)	0	0
Findlay I (1996) [5]	21/23 (91.3%)	2/23 (8.7%)	Poor delineation of cavity separation
Makrydimas G (1997) [6]	17/17 (100%)	0	0
Makrydimas G (2004) [7]	9/9 (100%)	0	0
Giambona A (2011) [8]	25/26 (96.2%)	1 (3.8%)	Inadequate coelomic fluid sample
Giambona A (2016) [9]	302/302 (100%)	0	0
Giambona A (2022) [10]	385/385 (100%)	0	0
Makrydimas G (2020) [11]	401/402 (99.8%)	1/402 (0.2%)	Not reported
Vinciguerra M (2022) [12]	1/1 (100%)	0	0
Giambona A (2024) [13]	5/5 (100%)	0	0
Makrydimas G (2002) [16]	106/107 (99.1%)	1/107 (0.9%)	Fibroid
Makrydimas G (1997) [17]	4/4 (100%)	0	0
Jouannic JM (2006) [18]	14/14 (100%)	0	0
Jouannic JM (2008) [19]	12/12 (100%)	0	0
Giambona A (2016) [20]	122/122 (100%)	0	0
Ross JA (1997) [21]	20/20 (100%)	0	0
Jauniaux E (2003) [22]	17/17 (100%)	0	0
Giambona A (2018) [23]	489/489 (100%)	0	0
Crüger DG (1997) [24]	1/1 (100%)	0	0
Makrydimas G (2004) [25]	20/20 (100%)	0	0
Chatzimeletiou K (2005) [26]	12/12 (100%)	0	0
Chatzimeletiou K (2006) [27]	1/1 (100%)	0	0
Giambona A (2022) [28]	13/13 (100%)	0	0
Makrydimas G (2006) [29]	13/13 (100%)	0	0
Tonni G (2009) [30]	1/1 (100%)	0	0
Tonni G (2009) [31]	1/1 (100%)	0	0
Tonni G (2016) [32]	1/1 (100%)	0	0
Aiello (2018) [33]	22/22 (100%)	0	0
Giambona A (2022) [34]	4/4 (100%)	0	0

**Table 3:** Diagnostic success rates stratified by object of testing.

Study	Genetic Test	Object of Testing	Diagnostic Success (n/N, %)	Type of Control
Findlay I (1996) [5]	QF-PCR	Single gene defects	19/23 (82.6%)	CVS
Makrydimas G (1997) [6]	PCR		4/4 (100%)	CVS
Makrydimas G (2004) [7]	QF-PCR		9/9 (100%)	POC, amnio, newborn
Giambona A (2011) [8]	Molecular analysis		25/25 (100%)	CVS, amnio, POC
Giambona A (2022) [10]	Molecular analysis		258/258 (100%)	POC
Makrydimas G (2020) [11]	Molecular analysis		376/376 (100%)	CVS, amnio, newborn, POC
Vinciguerra M (2022) [12]	Molecular analysis		1/1 (100%)	POC
Giambona A (2024) [13]	Molecular analysis		5/5 (100%)	CVS, amnio
Jouannic JM (2006) [18]	QF-PCR		8/14 (57.1%)	Maternal blood
Giambona A (2016) [20]	Molecular analysis		302/302 (100%)	CVS, amnio, newborn
Giambona A (2018) [23]	Molecular analysis		487/487 (100%)	CVS, amnio, newborn, POC
Makrydimas G (2004) [25]	QF-PCR		18/20 (90%)	CVS
Giambona A (2022) [28]	Molecular analysis		13/13 (100%)	CVS, newborn
Giambona A (2022) [34]	Molecular analysis		4/4 (100%)	CVS, amnio
Jurkovic D (1993) [1]	FISH	X, Y	10/10 (100%)	POC, CVS, amnio
Jurkovic D (1993) [1]	PCR		10/10 (100%)	POC, CVS, amnio
Findlay I (1996) [5]	QF-PCR		18/18 (100%)	CVS
Jouannic JM (2008) [19]	FISH		6/12 (50%)	Maternal blood
Aiello (2018) [33]	QF-PCR		22/22 (100%)	CVS, amnio
Jurkovic D (1993) [1]	Standard cytogenetics	X, Y, karyotype, or other autosomal chromosomes	0/10 (0%)	POC, CVS, amnio
Crüger DG (1996) [4]	Standard cytogenetics		9/9 (100%)	CVS
Findlay I (1996) [5]	Standard cytogenetics		9/9 (100%)	CVS
Jauniaux E (2003) [22]	QF-PCR		17/17 (100%)	CVS, maternal blood sample
Crüger DG (1997) [24]	Standard cytogenetics		1/1 (100%)	CVS
Chatzimeletiou K (2005) [26]	FISH		12/12 (100%)	CVS
Chatzimeletiou K (2006) [27]	FISH		1/1 (100%)	POC
Tonni G (2009) [30]	FISH		1/1 (100%)	POC
Tonni G (2009) [31]	Standard cytogenetics		1/1 (100%)	POC
Tonni G (2016) [32]	Standard cytogenetics		1/1 (100%)	POC

## Data Availability

Data are available from the corresponding author upon reasonable request.

## Abbreviations

The following abbreviations are used in this manuscript:

PCR	Polymerase Chain Reaction
FISH	Fluorescence In Situ Hybridization
CVS	Chorionic Villous Sampling
POC	Product of Conception
